# Moderate-intensity exercise alters markers of alternative activation in circulating monocytes in females: a putative role for PPARγ

**DOI:** 10.1007/s00421-016-3414-y

**Published:** 2016-06-23

**Authors:** J. S. Ruffino, N. A. Davies, K. Morris, M. Ludgate, L. Zhang, R. Webb, A. W. Thomas

**Affiliations:** 1 Centre for Biomedical Science, Cardiff Metropolitan University, Cardiff, CF5 2YB UK; 2 Centre for Endocrine & Diabetes Sciences, Cardiff University School of Medicine, Cardiff, CF14 4YU UK; 3 College of Medicine, Swansea University, Swansea, SA2 8PP UK

**Keywords:** Monocyte, Polarisation, Exercise, PPARγ, Insulin resistance

## Abstract

**Purpose:**

Monocytes may be primed towards differentiation into classically activated M1 macrophages or alternatively activated M2 macrophages. M1 macrophages greatly contribute to the inflammation which promotes insulin resistance, whereas M2 macrophages resolve inflammation. We have previously shown that exercise increases M2 marker expression in mixed mononuclear cells, possibly via activation of the nuclear transcription factor PPARγ. However, these effects have not been demonstrated specifically within monocytes. Thus, we aimed to investigate whether moderate-intensity exercise elicited similar effects on monocytic M1/M2 marker expression and PPARγ activity to those reported previously in mononuclear cells, so as to further elucidate the mechanisms by which exercise may alter inflammatory status and, accordingly, prevent insulin resistance.

**Methods/results:**

19 sedentary females completed an 8 week moderate-intensity exercise programme (walking 45 min, thrice weekly). Monocytes were isolated from blood via immunomagnetic separation; monocyte expression of M2 markers (Dectin-1: 2.6 ± 1.9-fold; IL-10: 3.0 ± 2.8-fold) significantly increased, whilst the expression of the M1 marker MCP-1 significantly decreased (0.83 ± 0.2 cf. basal), over the duration of the programme. Serum PPARγ activity levels and PPARγ target-genes (CD36: 1.9 ± 1.5-fold; LXRα: 5.0 ± 4.7-fold) were significantly increased after the 8 week exercise programme. Associated with these effects were significant improvements in systemic insulin sensitivity (McAuley’s ISI: Δ0.98 M/mU/L cf. basal).

**Conclusion:**

Exercise participation suppressed M1 markers and induced M2 markers in monocytes, potentially via PPARγ-triggered signalling, and these effects may contribute (perhaps via priming of monocytes for differentiation into M2 tissue-macrophages) to improved systemic insulin sensitivity in exercising participants. These findings provide an alternative mechanism by which exercise may exert its anti-inflammatory effects in order to prevent insulin resistance and type 2 diabetes.

**Electronic supplementary material:**

The online version of this article (doi:10.1007/s00421-016-3414-y) contains supplementary material, which is available to authorized users.

## Introduction

Insulin resistance is a prerequisite for the development of type 2 diabetes (T2D) (Defronzo and Tripathy 2009; Petersen et al. 2007) with its pathogenesis being strongly associated with the development of chronic local and systemic inflammation (Goldfine et al. 2013; Samuel and Shulman 2012). It has previously been shown that individuals with insulin resistance have a chronically elevated inflammatory status (Harford et al. 2011; Olefsky and Glass 2010), with some inflammatory mediators directly impairing insulin signalling by activating protein kinases to induce deleterious alternative phosphorylation of insulin signalling molecules (Olefsky and Glass 2010). Taken together, it is clear that chronic inflammation plays a key role in the development of insulin resistance and T2D.

Physical activity has long been known to aid in the prevention and management of insulin resistance, whereas a sedentary lifestyle is associated with increased risk of metabolic disease (Colberg et al. 2010). Several key studies have demonstrated that exercise training may prevent T2D incidence by approximately 50 %, either as a lone therapy (Pan et al. 1997) or in combination with other lifestyle interventions such as diet counselling or weight loss (Diabetes Prevention Program Research Group 2002; Eriksson et al. 1999). Interestingly, the effects of lifestyle modification appear to be stronger than those achieved with the pharmacological intervention of metformin (Knowler et al. 2002). As such, physical activity can play an important role in the prevention and management of T2D, and understanding the mechanisms by which physical activity improves insulin sensitivity may aid in the optimisation of effective non-pharmacological therapies. One mechanism by which exercise might improve insulin sensitivity may involve its impact on inflammation (Golbidi et al. 2012; Petersen and Pedersen 2005). For example, in response to exercise, muscle-derived IL-6 promotes elevated levels of anti-inflammatory cytokines, such as IL-10 and IL-1 receptor antagonist (IL-1Ra) (Febbraio and Pedersen 2002; Kristiansen and Mandrup-Poulsen 2005; Petersen and Pedersen 2005; Scheller et al. 2011), potentially inhibiting the actions of pro-inflammatory mediators to bring about benefits with regard to chronic inflammatory conditions (Golbidi et al. 2012; Febbraio and Pedersen 2002; Petersen and Pedersen 2005).

Macrophages are immune cells which exist in almost every tissue type (Geissmann et al. 2010). Under certain conditions, such as in acute inflammation, macrophage populations may be replenished by extravasation of circulating monocytes, which then differentiate into mature macrophages within tissues (Ginhoux and Jung 2014; Haldar and Murphy 2014). Macrophages exist in several different phenotypic states, depending on the stimuli to which they are exposed (Mantovani et al. 2004); for example, interferon-γ (IFN-γ; either alone or in combination with microbial ligands or other cytokines e.g., TNFα or MCP-1) gives rise to ‘classically activated’ M1 macrophages, which are characteristically pro-inflammatory, whilst IL-4 and/or IL-13 stimulation promotes the production of ‘alternatively activated’ M2 macrophages, which play regulatory and anti-inflammatory roles (Mantovani et al. 2004; Mosser and Edwards 2008; Sica and Mantovani 2012). It is thought that pro-inflammatory macrophages are major instigators of the inflammation which drives insulin resistance (Huang et al. 2010; Osborn and Olefsky 2012; Romeo et al. 2012). In support of this, macrophage-specific inhibition of the IKKβ or JNK pro-inflammatory pathways was found to protect against insulin resistance (Arkan et al. 2005; Han et al. 2013). Furthermore, it has been found that macrophages in individuals with obesity and/or T2D are skewed towards the M1 phenotype (Chinetti-Gbaguidi and Staels 2011; Lumeng et al. 2007; Pradhan Nabzdyk et al. 2013; You et al. 2013), while deletion of peroxisome proliferator activated receptor gamma (PPARγ), an important regulator of M2 polarisation, in murine macrophage cells promoted diet-induced systemic insulin resistance and/or glucose intolerance (Hevener et al. 2007; Odegaard et al. 2007). Exercise has also been shown to have a beneficial impact on immune cell function within tissues, and on local and systemic inflammation. For example, exercise training in obese individuals reduced systemic inflammation, specifically via decreased macrophage infiltration into adipose tissue (Bruun et al. 2006) whilst others have demonstrated a significant link between the influence of exercise on tissue-macrophages and the pathogenesis of insulin resistance (Ikeda et al. 2013; Kawanishi et al. 2010; Oliveira et al. 2013).

Interestingly, we have previously shown that exercise may upregulate PPARγ expression and activity in mixed peripheral mononuclear cells (PMNCs; cells which include monocytes) (Butcher et al. 2008; Thomas et al. 2012; Yakeu et al. 2010). We, and others, have also found that, following participation in exercise, macrophages and PMNCs appear to adopt a less inflammatory, M2-like phenotype (Ikeda et al. 2013; Oliveira et al. 2013; Yakeu et al. 2010). Since PPARγ has also been deemed ‘critical’ in priming monocytes for the M2 macrophage phenotype (Bouhlel et al. 2007), these findings suggest that the insulin-sensitising, anti-inflammatory effects of exercise may be attributed, in part to its ability to upregulate PPARγ-activity in monocyte/macrophages (Yakeu et al. 2010).

Therefore, the present research aimed to determine the effects of an 8 week, moderate-intensity exercise programme on expression of markers of M1 or M2 polarisation in isolated human monocytes, and to investigate the mechanisms behind any observed changes. We aimed to test the hypotheses that such an exercise programme brings about anti-inflammatory insulin-sensitising systemic effects within participants; that markers of M2 polarisation in primary human monocytes are induced following participation in the exercise programme; and that PPARγ signalling is increased by exercise, supporting a putative role for this signalling molecule in exercise-associated anti-inflammatory M2 macrophage polarisation.

## Materials and methods

### Participant recruitment

Exclusion criteria included; a physically active lifestyle [assessed using a short version International Physical Activity Questionnaire (IPAQ)], a history of cardiovascular disease (CVD) and any individuals on prescribed lipid-lowering or metabolism-altering drugs. A power calculation (Minitab v16) was used to determine that a sample size of at least 11 was required for this study. To account for a 25–30 % attrition rate, it was decided to recruit 19 healthy, yet sedentary participants onto the study (convenience sampling resulted in a female only cohort with a mean age of 42 ± 11 years). Informed consent was obtained from all participants and ethics was granted by the School of Health Sciences’ School Research Ethics Committee (SREC) at Cardiff Metropolitan University, Cardiff, UK.

### Pre-study screening/baseline measures

Body mass, height, BMI and waist circumference were measured using standard protocols (Butcher et al. 2008). Participant fitness was estimated using the submaximal Rockport Fitness Walking Test (Kline et al. 1987), where, following a 4 min familiarisation/warm up period, participants were required to walk as fast as possible for 1 mile (1.6 km) on a treadmill (Woodway Desmo, Waukesha, USA). Heart rate (HR) upon completion was measured using a Polar S410 HR monitor (Polar Electro, Finland) and time of completion was taken. VO_2max_ was estimated using the following formula (Kline et al. 1987):

$$ {\text{VO}}_{2\hbox{max} } = 132.853{-}\left( {0.0769 \, \left( {\text{body mass inpounds}} \right)} \right){-}\left( {0.3877 \, \left( {\text{age in years}} \right)} \right) + \left( {6.315 \, \left( {{\text{gender}};{\text{ male}} = 1,{\text{ female}} = 0} \right)} \right){-}\left( {3.2649 \, \left( {{\text{time to walk 1 mile in minutes}}/100{\text{ths of minutes}}} \right)} \right){-}\left( {0.1565 \, \left( {\text{heart rate upon mile completion in beats/minute}} \right)} \right). $$

Estimated VO_2max_ and anthropometric measures were taken at baseline and repeated upon completion of the exercise programme, ensuring a 24 h rest period between the final exercise session and the VO_2max_ test, in both cases.

### Exercise programme design

The exercise programme consisted of three 45 min walking sessions on a treadmill (Woodway Desmo, Waukesha, USA) per week for 8 weeks, totalling 24 fully supervised walking sessions. Treadmill walking speeds were set to ensure that participants exercised at moderate intensity (55–69 % of HRmax; HRmax = 220-age in years) (Bagchi and Preuss 2012). In subsequent sessions, HR was monitored using a Polar S410 HR monitor (Polar Electro, Finland) once weekly, at rest and then at 5, 15, 30 and 45 min into the exercise session. Throughout programme participation, walking speed was altered according to changes in HR to maintain a constant exercise intensity.

### Blood sampling

Participants were fasted for 12 h prior to blood collection by venipuncture of the antecubital vein. Blood was collected at four time points; immediately prior to (baseline; Wk 0, T0) and immediately following the first exercise session (Wk 0, T1) and immediately prior to (Wk 8, T0) and immediately following (Wk 8, T1) the final exercise session (T0 and T1 indicate pre- or post-exercise, respectively). A 24 h rest period was left between the penultimate exercise session and procurement of the ‘Wk 8, T0’ sample.

### Serum procurement

Whole blood was collected in plain blood tubes and allowed to clot prior to centrifugation for 10 min at 3000×*g*. Serum was stored in aliquots at −80 °C.

### Blood fractionation and monocyte purification

Whole blood was collected in EDTA and fractionated via density gradient centrifugation using Histopaque^®^-1077 (Sigma–Aldrich, Dorset, UK). The mononuclear cell layer was collected, washed in isotonic phosphate buffered saline (PBS) solution and centrifuged (300×*g*/10 min) to pellet mononuclear cells. An additional platelet wash (200×*g*/10 min) was carried out to remove contaminating platelets. Following this, monocytes were isolated via magnetic cell isolation using CD14 MACS MicroBeads (Miltenyi Biotec, Germany), as per manufacturer’s instructions, and monocyte purity was assessed using flow cytometry (see online resources ESM). Labelled cells were removed from the column and harvested by centrifuged.

### Biochemical analysis

Serum cholesterol, HDL, triglyceride and glucose levels were analysed, using an iLab 300 Plus analyser (Instrumentation Laboratories UK ltd, Warrington, UK). Serum LDL was quantified indirectly using the Friedewald (Friedewald et al. 1972) equation $\left( {\left[ {\text{LDL}} \right] = \left[ {\text{total cholesterol}} \right]{-}\left[ {\text{HDL}} \right]{-}\left( {\left[ {\text{triglycerides}} \right]/2.2} \right)} \right.$; all values expressed in mmol/l). Serum insulin was measured using the Invitrogen Insulin Assay Kit, as per manufacturer’s instruction (Invitrogen Ltd, Paisley, UK). The Berthold Technologies Centro Plate Luminometer (Herts, UK) was used to measure luminescence, whilst insulin concentrations were determined using MikroWin^®^ software.

### Calculation for insulin sensitivity

The McAuley’s score for measuring the insulin sensitivity index (McAuley’s ISI) was used as a surrogate measure of insulin resistance. This calculation has been found to be suitable for estimations of insulin resistance in normoglycaemic individuals, such as the participants within this study (Ascaso et al. 2003; McAuley et al. 2001). McAuley’s ISI was calculated as follows:

$$ {\text{McAuley's ISI}} = \exp \left[ {3.29 - 0.25 \times \ln ({\text{I0}}) - 0.22 \times \ln ({\text{BMI}}) - 0.28 \times \ln ({\text{TG}})} \right] $$

where I0 = fasting insulin (mU/l), BMI = body mass index (kg/m^2^), TG = fasting triglycerides (mmol/l).

### RNA isolation/RT-PCR

TRI reagent^®^ (Applied Biosystems, Warrington, UK) was used to obtain RNA from monocyte samples, as per manufacturer’s instructions. RNA quantity and quality were assessed using the NanoDrop^®^ ND-1000 spectrophotometer (Thermo Fisher Scientific, Leicester, UK) and RNA was converted into cDNA using a High Capacity cDNA Reverse Transcription kit (Applied Biosystems, Warrington, UK), as per manufacturer’s instructions. Gene expression was carried out on an Applied Biosystems Fast 7500 Real-Time PCR System (Warrington, UK) using Fast SYBR^®^ Green or TaqMan^®^ Fast Universal (No AmpErase^®^ UNG) methodologies. For Fast SYBR^®^ Green, primers were designed using National Center for Biotechnology Information’s ‘Primer-BLAST’ primer designing tool, and made to order (Sigma–Aldrich, Dorset, UK). The following primer sequences were used:

- Dectin-1. Fwd: 5′- GGAAGCAACACATTGGAGAATGG-3′; Rev: 5′- CTTTGGTAGGAGTCACACTGTC-3′
- IL-10. Fwd: 5′-ACGGCGCTGTCATCGATT-3′; Rev: 5′-TTGGAGCTTATTAAAGGCATTCTTC-3′
- IL-1Ra. Fwd: 5′-GGCCTCCGCAGTCACCTAATCAC-3′; Rev: 5′-GGACAGGCACATCTTCCCTCCAT-3′
- TNFα. Fwd: 5′-TGCCTGCTGCACTTTGGAGTGA-3′; Rev: 5′- CTGGGCCAGAGGGCTGATTAGAGA-3′
- CD36. Fwd: 5′-GGAAGTGATGATGAACAGCAGC-3′; Rev: 5′- GAGACTGTGTTGTCCTCAGCGT-3′
- LXRα. Fwd: 5′-CGCACTACATCTGCCACAGT-3′; Rev: 5′-TGAGGCGGATCTGTTCTTCT-3′
- ABCA1. Fwd: 5′-GCACTGAGGAAGATGCTGAAA-3′; Rev: 5′-AGTTCCTGGAAGGTCTTGTTCA-3′
- COX-2. Fwd: 5′-TGAAACCCACTCCAAACACA-3′; Rev: 5′-GAGAAGGCTTCCCAGCTTTT-3′
- GAPDH. Fwd: 5′-CATTGACCTCAACTACATG-3′; Rev: 5′-TCTCCATGGTGGTGAAGAC-3′.

Alternatively, TaqMan^®^ RT-PCR was used to determine MCP-1, IL-4 receptor α (IL-4Rα) and PPARγ gene expression. For TaqMan^®^ experiments, pre-designed PrimeTime^®^ Assays (Integrated DNA Technologies, Iowa, USA) which detected all genetic variants but not genomic DNA and those in which exon boundaries were consolidated were selected for use (assay configuration: 6-FAM/ZEN/IBFQ, P:P 2.0). A non-template control (NTC; nuclease free water instead of cDNA) was included for each gene analysed. The comparative C_T_ method was used to calculate gene expression relative to the reference sample. Data were only included if plots of log[RNA] versus ΔC_T_ resulted in slopes of between −0.1 and +0.1, meaning that amplicon efficiencies were approximately equal, and if plots of log template versus *C*_T_ gave slopes of approximately −3.3 (representing 100 % PCR efficiency). Baseline samples, i.e., those taken prior to the 8 week exercise programme, were used as reference samples.

### Gene reporter assays

Two gene reporter assays were conducted to investigate (1) the PPARγ activating properties of serum (‘PPRE-Luc’) and (2) PPARγ ligand availability (‘PPARγ-LBD’) in serum samples obtained from study participants. Human embryonic kidney (HEK-293T) cells (ATCC, Middlesex, UK) were maintained under standard conditions in DMEM (Invitrogen Ltd, Paisley, UK), supplemented with 10 % US Defined, Irradiated and Heat inactivated Fetal Bovine serum (FBS; Thermo Fisher Scientific, Leicester, UK), 100 U/mL penicillin/100 μg/mL streptomycin mix and 4 mM l-glutamine (Invitrogen Ltd, Paisley, UK). Cells were seeded in opaque 96-well plates at 2 × 10^5^ cells/well and incubated in a 37 °C, 5 % CO_2_, humidified incubator for 24 h prior to transient transfection.

### PPRE-Luc

Cells were transfected with 100 ng of each of the PPRE-luciferase reporter construct and the PPARγ expression vector and 2 ng of the *Renilla* plasmid, using Lipofectamine^®^ LTX Reagent (Invitrogen Ltd, Paisley, UK) according to manufacturer’s instructions and as previously described (Thomas et al. 2012). Cells were incubated with plasmids in a 37 °C, 5 % CO_2_, humidified incubator for a further 24 h, following which media was removed and replaced with plasma samples [normalised for protein content and added at 10 % (v/v) to DMEM (minus FBS)]. Alternatively cells were treated with 1 µM rosiglitazone (a known activator of PPARγ) as a positive control. After 24 h, cells were harvested and lysed and luminescence was analysed using the Dual-Luciferase^®^ Reporter Assay System (Promega, Southampton, UK) as per manufacturer’s instructions. Luminescence values were normalised with regard to transfection efficiency by use of a ratio of Luciferase:Renilla luminescence and reported as relative light units (RLU).

### PPARγ-LBD

Cells were transfected with 2 μg of a fusion vector carrying the PPARγ-LBD fused with the GAL4 DNA-binding domain (DBD) [PPARγ-LBD/GAL4-DBD; (Tzameli et al. 2004)], 2 μg of an Upstream Activation Sequence (UAS; GAL4 response element)-luciferase reporter construct [UAS; (Collingwood et al. 1997)] and 0.5 μg of β-gal, diluted in 1.5 mL unsupplemented DMEM and mixed with TransFast^TM^ Transfection Reagent (Promega, Southamptom, UK) in a 3:1 charge ratio of TransFast™ (μL) to DNA (μg). The TransFast™-plasmid mix was incubated for 10 min before cells were incubated with the plasmids in a 37 °C, 5 % CO_2_, humidified incubator for 1 h, before 2.5 mL of supplemented DMEM media (minus antibiotics) was added to cells. Cells were incubated with plasmids for a further 24 h, following which media was removed and replaced with plasma samples [normalised for protein content and added at 10 % (v/v) to DMEM (minus FBS)]. Alternatively cells were treated with 1 µM rosiglitazone (a known activator of PPARγ) as a positive control. After 24 h, cells were harvested and lysed and 40 μL of each lysate was transferred to the wells of an opaque 96 well plate and 50 μL of LAR II was auto-injected into each well and luminescence readings were taken using a Tecan Infinite 200 plate reader. Simultaneously, for internal control (β-gal) readings, 40 μL of each lysate was transferred into wells of a transparent 96-well plate and an equal volume of *o*-Nitrophynyl-beta-d-galactopyranosidase (ONPG; β-gal substrate) was added to wells, incubating for 30 min, or until a yellow colour developed. Absorbance was read using the Tecan Infinite 200 plate reader, set at 435 nm. The ratio of Luciferase luminescence to β-gal OD was used to normalise values, and values were reported as RLU.

### ELISA

Commercially available ELISA kits were used according to manufacturer’s instructions to determine serum cytokine levels; the Human IL-4 High Sensitivity ELISA (eBioscience, Vienna, Austria) was used to quantify IL-4 and The RayBio^®^ Human IL-13 ELISA Kit (Insight Biotechnology Ltd, Middlesex, UK) was used to quantify IL-13 in ‘Wk 0, T0’ and ‘Wk 8, T0’ serum samples, whilst the Quantikine^®^ HS ELISA Human IL-6 Immunoassay (R&D Systems, Abington, UK) was used to quantify serum IL-6 in ‘Wk 0, T0’ and ‘Wk 0, T1’ serum samples. Spectrophotometry was carried out on a Tecan Infinite 200 (Tecan, Reading, UK).

### Statistical analysis

All data were expressed as mean ± standard deviation (SD), unless otherwise stated. Where comparisons were made between the means of two samples, *t* tests or Wilcoxon’s pairwise analysis were used, depending on data distribution (the D’Agostino and Pearson omnibus normality test was used to test for normal distribution of data). Alternatively, one-way analysis of variance (ANOVA) with Tukey’s post hoc analysis was used for multiple comparisons within groups of normally distributed data. Statistical analysis was performed using GraphPad Prism^®^5 software and results were deemed significant at *p* < 0.05.

## Results

### The effect of an 8-week moderate-intensity walking programme on cardio-metabolic risk markers

Nineteen healthy, yet sedentary female participants were recruited onto the study (mean age of 42 ± 11 years; body weight 76.54 ± 12.54 kg; body mass index (BMI) 29.7 ± 5.1 kg/m^2^; waist circumference 91.74 ± 13.36 cm; systolic blood pressure 126.3 ± 9.9 mmHg; diastolic blood pressure 78.5 ± 8.8 mmHg. As shown in Table 1, mean body mass was significantly reduced following participation in the exercise intervention [*p* < 0.05 compared to baseline (‘Wk 0, T0’)], as was BMI (*p* < 0.05) and waist circumference (*p* < 0.01). Furthermore, systolic blood pressure showed a non-significant decrease (*p* = 0.059) and diastolic blood pressure was unaltered. Additionally, there was a significant increase in estimated VO_2max_ (*p* < 0.01).

**Table 1:** Physiological and biochemical parameters at baseline (Wk 0, T0) and after 8 weeks (Wk 8, T0) of exercise participation

	Wk 0, T0	Wk 8, T0	Δ (95 % CI)
Weight (kg)	76.5 ± 12.5	75.7 ± 12.4	−0.9 (−1.63, −0.09)*
BMI (kg/m^2^)	29.7 ± 5.1	29.4 ± 4.9	−0.4 (−0.67, −0.04)*
Waist circumference (cm)	91.7 ± 13.4	86.2 ± 12.0	−5.6 (−8.09, −3.05)**
Systolic BP (mmHg)	126.3 ± 9.9	121.2 ± 12.0	−5.1 (−10.36, −0.21)
Diastolic BP (mmHg)	78.4 ± 7.8	77.8 ± 10.9	−0.6 (−4.78, 3.52)
VO_2max_ (mL/kg/min)	26.8 ± 8.2	34.7 ± 5.3	−7.9 (4.95, 10.84)**
Total cholesterol (mmol/L)	4.8 ± 0.9	5.0 ± 1.1	−0.2 (−0.17, 0.47)
LDL (mmol/L)	2.6 ± 0.7	2.9 ± 0.8	−0.2 (−0.01, 0.49)
HDL (mmol/L)	1.6 ± 0.4	1.6 ± 0.4	−0.0 (−0.09, 0.07)
Triglycerides (mmol/L)	1.3 ± 0.5	1.1 ± 0.5	−0.2 (−0.34, −0.02)**
Fasting glucose (mmol/L)	4.8 ± 0.5	4.7 ± 0.5	−0.1 (−0.38, 0.15)
Fasting insulin (pmol/L)	49.9 ± 54.0	34.2 ± 19.9	−15.7 (−37.50, 6.12)
McAuley’s ISI (M/mU/L)	8.2 ± 2.2	9.1 ± 2.2	−0.98 (−0.0009, 1.96)*

Results are shown as mean ± SD with changes from baseline (Δ) presented with 95 % CI of the mean. *n* = 19 for all the cases except for VO_2max_, where *n* = 15 (for *n* = 4, baseline VO_2max_ levels were below the measurable range) and blood lipids and McAuley’s ISI, where *n* = 17 (for *n* = 2, blood lipid levels were undetectable). *p < 0.05, **p < 0.01, paired *t* test was used for all the analysis other than fasting insulin, glucose, and blood lipids, where Wilcoxon’s paired analysis was used

No significant changes were identified in serum total cholesterol, LDL or HDL. However, median levels of serum triglycerides were significantly reduced post intervention, when compared to baseline (‘Wk 0, T0’) (*p* < 0.01). Additionally, there was a significant increase in insulin sensitivity, as measured by McAuley’s ISI, when compared to baseline (*p* < 0.05, see Table 1).

### Markers of M1/M2 polarisation were significantly altered in monocytes following an 8-week moderate-intensity walking programme

**Fig. 1:**
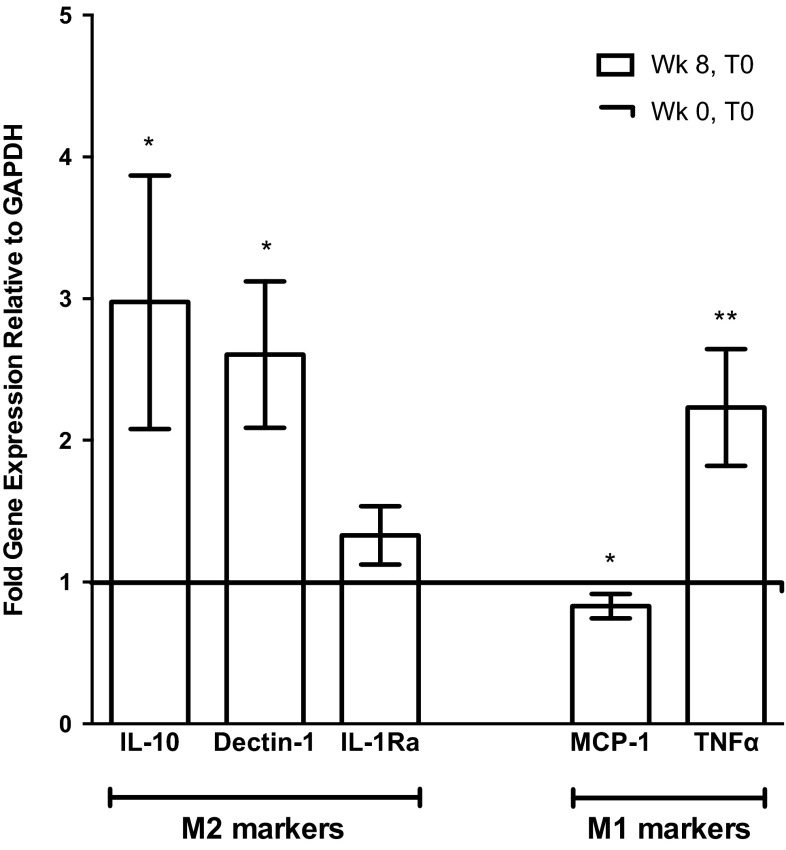
Effect of an 8 week, moderate-intensity exercise intervention on gene expression of M2 markers and M1 markers in human monocytes. Isolated human monocytes were obtained at baseline (Wk 0, T0) and prior to the final exercise session (Wk 8, T0) of an 8 week, moderate-intensity walking programme. Where possible, the baseline values for each individual were used as comparators. In some cases, RNA quality or quantity was not sufficient for use in RT-PCR or some genes of interest were expressed at levels below the acceptable range of the assay and so were excluded from analysis (IL-10, *n* = 10; Dectin-1, *n* = 14; IL-1Ra, *n* = 14; MCP-1, *n* = 8; TNFα, *n* = 14; values expressed as fold gene expression relative to GAPDH ± SEM; *p < 0.05, **p < 0.01, *t* test)

The M2 marker genes Dectin-1 and IL-10 were significantly upregulated by 2.6 ± 1.9-fold (*p* < 0.01) and 3.0 ± 2.8-fold, respectively (*p* < 0.05; Fig. 1). mRNA expression of IL-1Ra was also elevated by 1.3 ± 0.7-fold, showing a non-significant trend towards increased expression (*p* = 0.099). Conversely, the M1 marker, MCP-1, was significantly reduced to approximately 0.83 ± 0.2 (*p* < 0.05) of baseline (‘Wk 0, T0’) expression, following participation in the exercise intervention [in contrast, TNFα was significantly upregulated by 2.2 ± 1.4-fold (*p* < 0.01)].

### PPARγ activity was increased in monocytes following an 8-week moderate-intensity walking programme, potentially due to acute exercise-induced increases in the PPARγ-activating properties of serum

**Fig. 2:**
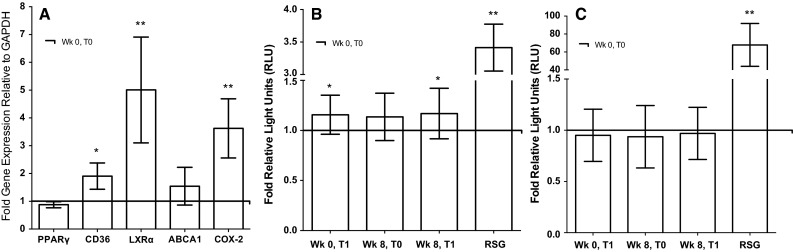
Effect of an 8 week, moderate-intensity exercise intervention on monocyte expression/activity of PPARγ and mechanisms for PPARγ activation. **a** Monocytes were obtained at baseline (Wk 0, T0) and immediately prior to the final exercise session (Wk 8, T0) of an 8 week, moderate-intensity walking programme and gene expression of PPARγ and PPARγ-associated genes, CD36, LXRα, ABCA1, and COX-2, was analysed. The baseline values for each individual were used as comparators. Gene reporter assays were used to analyse **b** the PPARγ activating properties of serum and **c** PPARγ ligand availably in serum obtained at baseline (Wk 0, T0), immediately following the initial exercise bout (Wk 0, T1), immediately prior to the final exercise bout (Wk 8, T0), and immediately following the final exercise bout (Wk 8, T1) of an 8 week, moderate-intensity exercise intervention. Rosiglitazone (RSG) was used as positive control luminescence values that were normalised to an internal control vector to obtain RLU values corresponding to PPARγ activity (*RT*-*PCR:* PPARγ, *n* = 10; LXRα and ABCA1, *n* = 9; CD36, *n* = 16; COX-2, *n* = 12; values expressed as fold gene expression relative to GAPDH ± SEM, *p < 0.05, **p < 0.01, *t* test. Gene reporter assays: *n* = 19 for serum, *n* = 3 for RSG; values expressed as fold RLU ± SD, *p < 0.05, **p < 0.01, one-way ANOVA)

The effects of exercise on PPARγ were investigated to provide a potential mechanism for the increase in M2 marker expression observed in monocytes in response to the exercise intervention. Although PPARγ mRNA expression did not alter significantly in monocytes following exercise training, when compared to baseline (‘Wk 0, T0’) (Fig. 2a), monocytic expression of the PPARγ-regulated downstream target genes, CD36 (1.9 ± 1.5-fold, *p* < 0.05), Liver X receptor α (LXRα; 5.0 ± 4.7-fold, *p* < 0.01), and ABCA1 (1.5 ± 1.9-fold, *p* > 0.05) was increased (Fig. 2a). In addition, the expression of cyclooxygenase-2 (COX-2), an enzyme associated with the generation of intracellular PPARγ ligands (Díaz-Gandarilla et al. 2013), was also significantly upregulated in monocyte samples obtained prior to the final exercise session, when compared to baseline (‘Wk 0, T0’) (3.6 ± 2.7-fold, *p* < 0.01; Fig. 2a).

To investigate potential mechanisms for exercise-induced PPARγ activation, gene reporter assays were used to analyse the PPARγ-activating properties of serum and PPARγ ligand generation in serum samples obtained throughout the exercise study. Figure 2b demonstrates that the PPARγ-activating properties of serum (as measured using PPRE-luciferase reporter assay) were significantly increased compared to baseline (‘Wk 0, T0’) immediately following individual exercise bouts (‘Wk 0, T1’: 1.16 ± 0.19-fold RLU; ‘Wk 8, T1’: 1.17 ± 0.25-fold RLU,-fold values relative to baseline (‘Wk 0, T0’), *p* < 0.05), while the PPARγ activating properties of serum underwent a non-significant increase over the duration of the entire exercise training programme [‘Wk 8, T0’: 1.14 ± 0.24-fold RLU, fold relative to baseline (‘Wk 0, T0’)]. Interestingly, however, neither acute exercise nor exercise training significantly altered ligand generation, as measured by the PPARγ-LBD gene reporter assay, [Fig. 2c; ‘Wk 0, T1’; 1.09 ± 0.26-fold RLU; ‘Wk 8. T0’: 0.94 ± 0.30-fold RLU; ‘Wk 8, T1’: 0.97 ± 0.26-fold RLU, fold values relative to baseline (‘Wk 0, T0’)].

### Elevations in IL-6 were observed after individual exercise bouts; however, alterations in IL-4 or IL-13 were not detected over the duration of an 8-week moderate-intensity walking programme

**Fig. 3:**
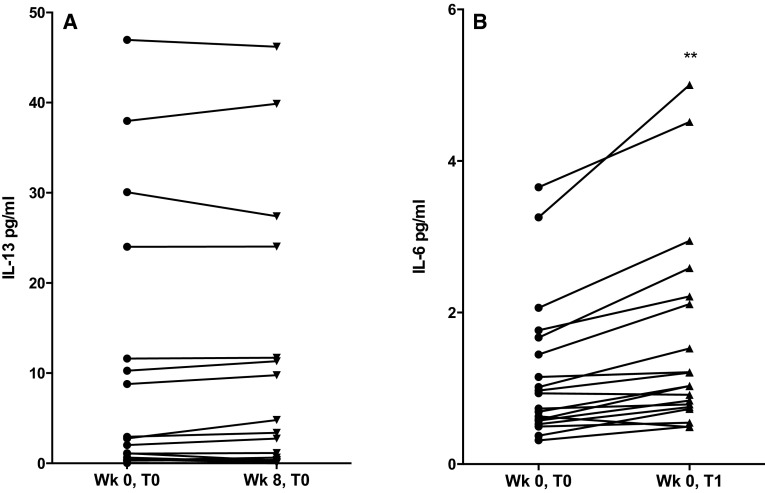
Serum IL-13 and IL-6 concentration in response to an 8 week, moderate-intensity brisk walking intervention. **a** Serum IL-13 and **b** serum IL-6 were measured via ELISA. Serum IL-13 concentration was measured in samples taken at baseline (Wk 0, T0) and immediately prior to the final bout of an 8 week, moderate-intensity exercise intervention (Wk 8, T0), whereas serum IL-6 was measured in samples taken at baseline (Wk 0, T0) and following an acute bout of moderate-intensity exercise (Wk 0, T1) [IL-13, *n* = 17 (for *n* = 2, IL-13 levels were below the detection limit of the assay); IL-6, *n* = 19; results expressed as individual data points; significance related to mean values; **p < 0.01, paired *t* test]

In the present study, levels of the M2-promoting stimulus, IL-4, were non-detectable in serum samples obtained either at baseline (‘Wk 0, T0’) or at the final exercise session (‘Wk 8, T0’) (minimum detection levels of the assay were 0.25 pg/mL; data not shown), whilst serum levels of another M2 stimuli, IL-13, did not alter significantly over the course of the exercise programme (‘Wk 0, T0’: 10.52 ± 3.43 pg/mL; Wk 8, T0: 11.20 ± 3.47 pg/mL; see Fig. 3a). Serum IL-6 concentrations increased significantly immediately after individual exercise bouts (‘Wk 0, T0’: 1.20 ± 0.21 pg/mL vs ‘Wk 0, T1’: 1.63 ± 1.32 pg/mL, *p* < 0.01, see Fig. 3b); however, baseline (‘Wk 0, T0’) levels of serum IL-6 were not altered over the duration of the 8 week exercise programme (results not shown).

## Discussion

Previously, our group has demonstrated an increased M2 marker expression in human PMNCs following participation in an 8 week walking intervention (Yakeu et al. 2010). Since monocytes may be ‘primed’ for differentiation into specific M1 or M2 macrophage states (Bouhlel et al. 2007), it was deemed important to determine whether the previously observed effect of exercise on M2 marker expression occurs specifically within monocytes. This study demonstrates that participation in an 8 week, moderate-intensity brisk walking intervention upregulated markers of alternative activation in circulating monocytes and improved systemic insulin sensitivity, and provided evidence for a role for PPARγ as a putative mediator of these exercise-induced effects.

Specifically, the monocyte M2 markers, IL-10, Dectin-1, and IL-1Ra, were found to increase following the exercise intervention (see Fig. 1); mRNA expression of IL-10 and Dectin-1 was significantly elevated, whereas the increase in IL-1Ra expression approached significance. In contrast, the gene expression of the M1 marker, MCP-1, significantly decreased to ~0.8-fold of basal levels, which is in agreement with the previous findings (Kawanishi et al. 2010; Oliveira et al. 2013; Yakeu et al. 2010). However, the expression of the M1 marker, TNFα, was significantly elevated by ~twofold (Fig. 1). Interestingly, the profile of polarisation markers expressed by monocytes following exercise training in the current study is representative of a specific M2 macrophage subtype; the ‘M2b’ macrophage subset, which are said to produce M1-associated cytokines (including TNFα) but also high levels of IL-10, causing M2b cells to be immuno-regulatory (Hao et al. 2012; Kharraz et al. 2013; Mantovani et al. 2004). However, additional research would be required to substantiate the speculation that exercise training drives monocytes into an M2b phenotype. To our knowledge, this study is the first to demonstrate that exercise training increases markers of M2 polarisation specifically within monocytes, in a way which may drive them towards differentiation into the M2 macrophage phenotype, regarded as beneficial in the prevention of insulin resistance and T2D (67). However, at this stage, it should not be assumed that the significant improvement observed in insulin sensitivity (Δ0.98 M/mU/L compared to baseline, *p* < 0.05) in this study was solely a result of the changes in monocytic expression of M1/M2 markers in the present study (Table 1). Furthermore, it is well known that moderate exercise significantly lowers triglycerides, as was the case in this study, and this may also be an important contributing factor to the observed improvement in insulin sensitivity (Kraus and Slentz 2009).

We have previously shown that both acute and chronic exercise may induce the activation of PPARγ, a transcription factor thought to play an important role in priming monocytes for M2 polarisation (Bouhlel et al. 2007; Butcher et al. 2008; Thomas et al. 2012; Yakeu et al. 2010). However, this has only been demonstrated in mixed mononuclear cells (Butcher et al. 2008; Thomas et al. 2012; Yakeu et al. 2010), and has not yet been investigated within isolated monocytes. The findings from the current study suggest that PPARγ activity was elevated in monocytes in response to exercise training: PPARγ downstream genes, namely, CD36, LXRα and COX-2, were significantly upregulated after the 8 week intervention when compared to baseline, while ABCA1 underwent a non-significant increase (see Fig. 2a). CD36 is also associated with the M2 macrophage phenotype (Bouhlel et al. 2007; Oh et al. 2012) and so its upregulation may be used to further support the impact of exercise on monocyte activation.

To further investigate the mechanism(s) by which exercise may have enhanced monocyte PPARγ activity, both serum PPARγ activating properties and serum PPARγ ligand availability were determined using gene reporter assays. The PPARγ-activating properties of serum were shown to increase acutely upon exercise (Fig. 2b), despite no changes in PPARγ expression (Fig. 2a) or serum PPARγ ligand availability in response to acute or chronic physical activity (Fig. 2c). These results are in accordance with those we obtained previously and provide further evidence that exercise promotes PPARγ activation (Thomas et al. 2012). While the serum ligand availability assays demonstrate that this effect is independent of exogenous ligand production, our data suggest that exercise may instead trigger endogenous PPARγ ligand generation, as exercise-associated elevations in monocyte COX-2 expression (Fig. 2a) may contribute to increased PPARγ activity within monocytes, since COX-2 encodes an inducible enzyme which is responsible for the production of endogenous PPARγ ligands (e.g., 15d-PGJ_2_) (Díaz-Gandarilla et al. 2013; Heusinkveld et al. 2011; Sica and Mantovani 2012). In addition, exercise participation may trigger ligand-independent post-translational modifications of PPARγ, such as phosphorylation or sumoylation, resulting in PPARγ activation (Harmon et al. 2011; Olefsky and Glass 2010). However, it is a limitation of the current study that the specific PPARγ activating factor(s) responsible for this effect was not identified. In addition, the model system used in these experiments (HEK293-T cells) may not express the relevant ligand uptake/processing machinery, and thus, the in vivo situation may not be accurately reflected by these results. Future work would benefit from using cells which more closely resemble human monocytes.

It is also possible that exercise-induced alterations in systemic cytokine levels may impact upon PPARγ activity and M1/M2 marker expression in monocytes. Despite no observed change in serum levels of the established M2-stimuli IL-4 (data not shown) and IL-13 (Fig. 3a) (Mantovani et al. 2004) following exercise training, IL-6 was found to be significantly elevated in serum immediately following participation in exercise bouts (Fig. 3b). IL-6 is known to be synthesised by contracting muscle during acute exercise (Pan et al. 2012; Dishman et al. 2013); accordingly serum levels of IL-6 are transiently elevated upon exercise participation (Pedersen and Febbraio 2008), rapidly returning to baseline within 1–2 h of exercise cessation (Gleeson et al. 2011; Moldoveanu et al. 2000). Recently, an interesting role for IL-6 in the alternative activation of monocytes has been revealed: Mauer et al. demonstrated that IL-6 is required for IL-4-dependent M2 macrophage polarisation, and that the depletion of the IL-6Rα chain of the IL-6 receptor in myeloid cells induced insulin resistance and impaired glucose homeostasis (Mauer et al. 2014). Moreover, IL-6 was shown to upregulate IL-4 receptor (IL-4Rα) expression in macrophages, making these cells more sensitive to IL-4 stimulation. In addition, Szanto et al. (2010) demonstrated that IL-4/STAT-6 signalling resulted in augmented PPARγ activation in macrophages independent of changes in ligand availability. Thus, it may be speculated that in the present study, exercise IL-6-induced upregulation of IL-4Rα and subsequent increased sensitivity to IL-4 may contribute to the observed exercise-associated monocyte PPARγ activation (Fig. 2c), independent of changes in PPARγ ligand availability.

We acknowledge that a major limitation of this research is its reliance on the assumption that the M2 monocyte phenotype is retained upon extravasation and differentiation into tissue macrophages, whereas the possibility of intra-tissue ‘switching’ of macrophage phenotype has been previously reported (Dalmas et al. 2011; Lee et al. 2013). Thus, further investigations are required to elucidate the effects of similar exercise interventions on M1:M2 ratio in various tissue environments. Nevertheless, it should be noted that, if a link can be made between the classification of monocytes and that of macrophages, the current research may facilitate the use of monocytes (which are easily accessible cells) as biomarkers for macrophage-associated diseases, such as T2D.

Importantly, regardless of the specific PPARγ-activating process/es, exercise-associated increases in PPARγ-activating properties of serum may contribute to the systemic beneficial effects of the current exercise programme with regard to insulin sensitivity (Table 1). We propose that, in a similar way to PPARγ-targeting anti-diabetic agents, exercise participation may increase levels of blood-borne PPARγ-activating factors, allowing for sustained systemic PPARγ activation in a range of cells and tissues, and this may, together with many other contributing factors, impact upon glucose metabolism, lipid homeostasis, and inflammation within these tissues (Harmon et al. 2011; Wahli and Michalik 2012). In addition, we would contend that these findings further support the use of exercise prescription as an alternative to currently available pharmacological therapies with regard to the management and treatment of T2D and its cardiovascular complications.

## Conclusions

In conclusion, participation in an 8 week, moderate-intensity exercise programme induces M2 marker expression and PPARγ activation in monocytes (potentially priming them for differentiation into M2-polarised tissue macrophage) and exerts beneficial systemic effects with regard to insulin resistance. In addition to the observed beneficial alterations in key predictors of cardio-metabolic risk, including body weight, BMI, waist circumference, triglycerides, aerobic capacity, and insulin sensitivity, the current study provides further evidence that the prescription of a moderate-intensity walking programme may be beneficial to the prevention of T2D and its cardiovascular complications in at risk populations.

## Electronic supplementary material

Below is the link to the electronic supplementary material.

Supplementary material 1 (DOCX 163 kb)

## Abbreviations

ABCA1: ATP-binding cassette transporter
COX2: Cyclooxygenase 2
CD36: Cluster of differentiation 36
GAPDH: Glyceraldehyde 3-phosphate dehydrogenase
IL: Interleukin
ISI: Insulin sensitivity index
LXRα: Liver x receptor alpha
MCP-1: Macrophage chemotactic protein-1
PPARγ: Peroxisome proliferator activated receptor gamma
RLU: Relative light units
TNFα: Tumour necrosis factor alpha
T2D: Type 2 diabetes
