# Supplementary material for: Moderate-intensity exercise alters markers of alternative activation in circulating monocytes in females: a putative role for PPARγ
Source: Eur J Appl Physiol. 2016 Jun 23;116:1671–82. doi: 10.1007/s00421-016-3414-y (PMC4983283; doi:10.1007/s00421-016-3414-y)
Supplement: Supplementary file 1 — Supplementary material 1 (DOCX 163 kb) [file 421_2016_3414_MOESM1_ESM.docx]

**Supplementary material:**

Isolation of monocytes from mixed leukocyte populations: Monocytes were isolated via magnetic cell isolation using CD14 MACS MicroBeads as per manufacturer’s instructions. Monocyte purity was assessed via flow cytometry, using FITC-conjugated antibodies for the monocyte marker CD14 with IgG2a isotype control (Beckman Coulter, High Wycombe, UK). Findings confirmed that use of this technique resulted in a significant increase in monocyte content within samples (untreated leukocytes [red trace on Fig 4]: 8.7±4.1% CD14^+^ monocytes *vs.* isolated monocytes [blue trace on Fig 4]: 45.5 ± 9.0% CD14^+^ monocytes; *p*<0.01).


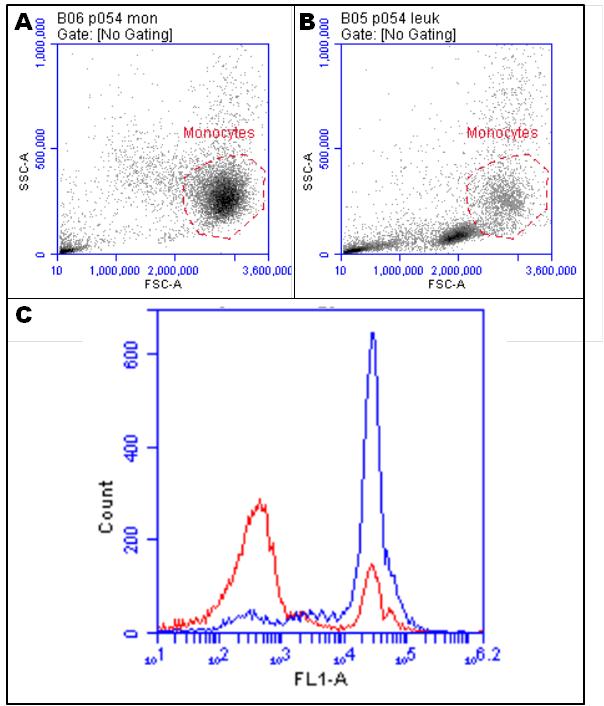


# Fig 4 Flow cytometric confirmation of monocyte isolation Flow cytometry using CD14-FITC antibody with IgG2a isocontrol (Beckman Coulter, High Wycombe, UK) was used to obtain scatter plots for immuno-magnetically isolated cells (a) and untreated leukocytes (b), and differential cell counts (c) for untreated leukocytes (red trace) and immunomagnetically isolated cells (blue trace).
